# Early Prostate Cancer Deaths Among Men With Higher vs Lower Genetic Risk

**DOI:** 10.1001/jamanetworkopen.2024.20034

**Published:** 2024-07-03

**Authors:** Anna Plym, Yiwen Zhang, Konrad H. Stopsack, Emilio Ugalde-Morales, Tyler M. Seibert, David V. Conti, Christopher A. Haiman, Aris Baras, Tanja Stocks, Isabel Drake, Kathryn L. Penney, Edward Giovannucci, Adam S. Kibel, Fredrik Wiklund, Lorelei A. Mucci

**Affiliations:** 1 Department of Medical Epidemiology and Biostatistics, Karolinska Institutet, Stockholm, Sweden; 2 Department of Epidemiology, Harvard T. H. Chan School of Public Health, Boston, Massachusetts; 3 Department of Urology, Brigham and Women’s Hospital, Harvard Medical School, Boston, Massachusetts; 4 Clinical and Translational Epidemiology Unit, Massachusetts General Hospital and Harvard Medical School, Boston; 5 Department of Radiation Medicine and Applied Sciences, Department of Radiology, and Department of Bioengineering, University of California San Diego, La Jolla; 6 Center for Genetic Epidemiology, Department of Population and Public Health Sciences, Keck School of Medicine, University of Southern California, Los Angeles; 7 Regeneron Genetics Center, Tarrytown, New York; 8 Department of Translational Medicine, Lund University, Malmö, Sweden; 9 Department of Clinical Sciences in Malmö, Lund University, Malmö, Sweden; 10 Skåne University Hospital, Malmö, Sweden; 11 Channing Division of Network Medicine, Department of Medicine, Brigham and Women’s Hospital and Harvard Medical School, Boston, Massachusetts; 12 Department of Nutrition, Harvard T.H. Chan School of Public Health, Boston, Massachusetts

## Abstract

**Importance:**

Prostate cancer, a leading cause of cancer death among men, urgently requires new prevention strategies, which may involve targeting men with an underlying genetic susceptibility.

**Objective:**

To explore differences in risk of early prostate cancer death among men with higher vs lower genetic risk to inform prevention efforts.

**Design, Setting, and Participants:**

This cohort study used a combined analysis of genotyped men without prostate cancer at inclusion and with lifestyle data in 2 prospective cohort studies in Sweden and the US, the Malmö Diet and Cancer Study (MDCS) and the Health Professionals Follow-Up Study (HPFS), followed up from 1991 to 2019. Data were analyzed between April 2023 and April 2024.

**Exposures:**

Men were categorized according to modifiable lifestyle behaviors and genetic risk. A polygenic risk score above the median or a family history of cancer defined men at higher genetic risk (67% of the study population); the remaining men were categorized as being at lower genetic risk.

**Main Outcomes and Measures:**

Prostate cancer death analyzed using time-to-event analysis estimating hazard ratios (HR), absolute risks, and preventable deaths by age.

**Results:**

Among the 19 607 men included for analysis, the median (IQR) age at inclusion was 59.0 (53.0-64.7) years (MDCS) and 65.1 (58.0-71.8) years (HPFS). During follow-up, 107 early (by age 75 years) and 337 late (after age 75 years) prostate cancer deaths were observed. Compared with men at lower genetic risk, men at higher genetic risk had increased rates of both early (HR, 3.26; 95% CI, 1.82-5.84) and late (HR, 2.26; 95% CI, 1.70-3.01) prostate cancer death, and higher lifetime risks of prostate cancer death (3.1% vs 1.3% [MDCS] and 2.3% vs 0.6% [HPFS]). Men at higher genetic risk accounted for 94 of 107 early prostate cancer deaths (88%), of which 36% (95% CI, 12%-60%) were estimated to be preventable through adherence to behaviors associated with a healthy lifestyle (not smoking, healthy weight, high physical activity, and a healthy diet).

**Conclusions and Relevance:**

In this 20-year follow-up study, men with a genetic predisposition accounted for the vast majority of early prostate cancer deaths, of which one-third were estimated to be preventable. This suggests that men at increased genetic risk should be targeted in prostate cancer prevention strategies.

## Key Points

**Question:**

What is the difference in risk of early prostate cancer death between men at higher vs lower genetic risk?

**Findings:**

In this cohort study of 19 607 men, men at higher genetic risk had a 3-fold increased risk of an early prostate cancer death. Thirty-six percent of the deaths in this group were estimated to be preventable through factors that are associated with a healthy lifestyle.

**Meaning:**

These findings suggest that targeting men at increased genetic risk with prevention strategies may substantially reduce the number of early deaths from prostate cancer.

## Teaser

This cohort study evaluates risk of early prostate cancer death among men with higher vs lower genetic risk.

## Introduction

Despite substantial progress in early detection and treatment for prostate cancer over the past decade, prostate cancer remains one of the leading causes of cancer death among men.^1^ Each year, approximately 400 000 men die from prostate cancer globally. Although prostate cancer is generally diagnosed among older men, approximately one-third of men who died from prostate cancer died before the age of 75 years, highlighting that the burden of early prostate cancer death is high and that new prevention strategies are needed.

One such strategy could involve targeting of high-risk populations such as men with a high polygenic risk score (PRS). PRSs of common genetic variants provide robust risk stratification for prostate cancer incidence^2,3,4^ as well as prostate cancer mortality^5,6^ across different populations and ancestry groups. Men in the top decile of PRSs have a lifetime prostate cancer risk of around 40% to 50%,^2,4^ and a 4-fold increased rate of prostate cancer death compared with men with a low PRS.^5^ Studies including family history of cancer^7,8^ or with direct measurements of specific rare variants^9,10^ show that such information can capture additional risk not captured by PRS. PRSs for prostate cancer have not yet been widely implemented in any country, but initial work supports their use in better identification of men at risk for potentially aggressive prostate cancer.^11^

In addition to early detection and treatment of premetastatic cancers in the high-risk population, a further strategy could involve stronger promotions of healthy lifestyle choices. While the established risk factors for prostate cancer (age, family history, race and ethnicity, height, and genetic factors) are nonmodifiable, multiple lines of evidence suggest that the risk of prostate cancer progression and death could be reduced through lifestyle behaviors such as not smoking, maintaining a healthy weight, and regular physical activity.^12,13,14^ Initial data suggest that this risk reduction for prostate cancer death may be particularly relevant for high-risk populations, such as men with a high PRS.^6^

It is unclear to what extent a prevention strategy targeted at men with high genetic risk can reduce premature death from prostate cancer. We addressed this question by analyzing data from 2 prospective cohort studies, the Malmö Diet and Cancer Study (MDCS) and the Health Professionals Follow-Up Study (HPFS). We focused on prevention through modifiable factors operationalized through a healthy lifestyle score, which was available for men in both cohorts, before potential prostate cancer diagnoses.

## Methods

### Study Population

The MDCS is a population-based prospective cohort study of 30 446 women and men from Malmö, Sweden, recruited between 1991 and 1996 aged 46 to 73 years.^15^ Participants filled in lifestyle questionnaires^16^ and had anthropometric measures recorded and blood drawn by a study nurse at baseline. A total of 12 120 men were recruited to the study, of whom 11 643 have been successfully genotyped. The study population for this analysis was restricted to genotyped men without a prostate cancer diagnosis at baseline and who had lifestyle data available (10 270 men).

The HPFS is a prospective cohort study of 51 529 US male health professionals, recruited in 1986, aged 40 to 75 years.^17^ At baseline and biennially during follow-up, participants filled in questionnaires collecting data on lifestyle, medications, and disease outcomes. Blood was collected between 1993 and 1999 (18 159 participants), and buccal cells between 2005 and 2006 (13 956 additional participants). Of these men, 10 917 had genotype data available through previously performed nested case-control studies.^18^ For this analysis, we included genotyped men alive and prostate cancer free in 1996, when information on family history of prostate and breast cancer was collected, who had complete lifestyle data (9337 men).

All participants in each study gave written informed consent, and the protocol for this cohort study was approved by the regional ethical review board in Lund, Sweden (MDCS) and the institutional review boards of Brigham and Women’s Hospital and Harvard T.H. Chan School of Public Health, and those of participating registries as required (HPFS). This report follows the Strengthening the Reporting of Observational Studies in Epidemiology (STROBE) reporting guideline for observational studies.

### Definition of a Healthy Lifestyle

We defined a healthy lifestyle based on a previously published lifestyle score for lethal prostate cancer^6,19^ (eTable 1 in Supplement 1). The following lifestyle factors were included: not smoking, having a healthy weight, high physical activity, and 3 components of a healthy diet (high intakes of tomato-based products, high intake of fatty fish, and low intake of processed meat). As in the original publication,^19^ the factors were combined into a score (1 point was assigned for each factor) with a healthy lifestyle was defined as a score of 3 to 6 and an unhealthy lifestyle as a score of 0 to 2. We also included a more detailed 4-group lifestyle categorization, in which the healthy group was defined as nonsmokers with a BMI less than 30, a high physical activity level, and a healthy diet (fulfilling ≥2 of the dietary components). In a sensitivity analysis, we constructed a healthy diet indicator using different dietary components, either fulfilling 4 or more of the World Cancer Research Fund and American Institute of Cancer Research (WCRF/AICR) dietary recommendations^20,21,22^ or fulfilling 2 or more dietary components from a 2022 literature review^23^ with a focus on factors associated with a reduced risk of more aggressive prostate cancer: high fiber intake (a component in the anti-inflammatory and antihyperinsulinemia diet), low processed meat intake, and low dairy product intake (eTable 1 in Supplement 1).

### Definition of Genetic Risk

We defined genetic risk based on a previously developed multiancestry PRS for overall prostate cancer^24^ including 400 genetic risk variants, of which 384 were available in both cohorts. This 400-variant PRS excludes 51 variants linked to prostate-specific antigen (PSA) levels and was in the original publication associated with aggressive prostate cancer in several ancestry groups, including men of European ancestry who were examined in the present analysis (genotyped men in both cohorts are nearly exclusively men of European decent, based on self-reported information). In our data, the 400-variant PRS provided a marginally stronger risk discrimination for early prostate cancer death than a 451-variant PRS (eTable 2 in Supplement 1). The PRS was calculated based on genotyped data,^18,25^ imputed using the 1000 genomes reference panel. The definition of a positive family history of cancer was dependent on the data available and was defined as any cancer (MDCS) or prostate or breast cancer (HPFS) in at least 1 first-degree relative. Based on prior work, we defined a higher genetic risk as having a PRS above the median or a family history of cancer, with the remaining men categorized as being at lower genetic risk.^7^

### Definition of the Outcome

The outcome was prostate cancer-specific death, collected through a linkage with the Swedish Cause of Death Register (MDCS) or through searches in the National Death Index and reports of next of kin, with causes of death assigned by a physician committee blinded to exposure data (HPFS). Based on prior work,^7^ we defined early prostate cancer deaths as those occurring by age 75 years and late prostate cancer deaths as those occurring after age 75 years. Lifetime risk refers to deaths occurring by age 85 years.

### Statistical Analysis

All men were followed up from the date of DNA collection or from 1996 (HPFS) until the date of prostate cancer death, death from other causes, emigration (MDCS), or end of follow-up, which was January 1, 2019 (HPFS), or December 31, 2019 (MDCS). Because men were followed up through national registers, loss to follow-up can be expected to be minimal.

We calculated hazard ratios (HRs) and 95% CIs for the association between genetic and lifestyle factors and prostate cancer death using Cox regression with age as the underlying time scale (left truncated at the age at start of follow-up) and with stratification by 10-year birth cohort (allowing for different baseline hazards). To take potential sampling bias from the genetic sampling in the HPFS into account, models for this cohort were fitted using inverse-probability weighted Cox regression models as previously described.^6^ Adjustments were made for calendar year of inclusion, education (MDCS), PSA screening history (HPFS), principal components (PCs) 1 to 3 of genetic variation (HPFS), personal history of other cancers, history of diabetes, aspirin use, statin use, and total energy intake. We examined early and late prostate cancer deaths through splitting the follow-up time into 2 categories, up until age 75 years and after age 75 years. Lifestyle factors were further examined in analyses stratified by the 2 genetic risk groups. We pooled estimates from the 2 cohorts using fixed-effects meta-analysis.

We further calculated age-specific absolute risks (cumulative incidence) of prostate cancer death using regression standardization within a flexible parametric framework,^26^ with death from other causes treated as a competing risk and standardizing over the same covariates as described previously. Based on the estimated average cumulative incidence by ages 70, 75, 80, and 85 years, we calculated the proportion of prostate cancer deaths that would have been prevented had everyone adhered to a healthy lifestyle at study entry, the attributable fraction (AF). This was estimated based on the following formula:

AF = 1 − [Pr(*Y*_0_ = 1)] / [Pr(*Y* = 1)].

Pr(*Y* = 1) is the factual probability of the outcome in the whole population and Pr(*Y*_0_ = 1) is the counterfactual probability of the outcome if the exposure was eliminated.^27^ These calculations were based on the exact probabilities (not rounded). We calculated 95% CIs to show the plausible range of estimates given the data and to allow for examinations of statistical significance corresponding to a 2-sided value of *P* < .05. Analyses were performed in R versions 4.2.3 and 4.3.1 (R Project for Statistical Computing) and Stata versions 17 and 18 (Stata Corp). Data were analyzed from April 2023 to April 2024.

## Results

Among the 19 607 men included for analysis, the median age at start of follow-up was 59.0 (53.0-64.7) years (MDCS) and 65.1 (58.0-71.8) years (HPFS) (Table 1). Combining the PRS and family history of cancer, 13 186 men (67%; 7413 [72%] in the MDCS and 5773 [62%] in the HPFS) were categorized as being at higher genetic risk. A total of 3048 men (30%) in the MDCS and 2692 (29%) in the HPFS had a lifestyle score of 0 to 2.

**Table 1.** Characteristics of Men in the Malmö Diet and Cancer Study (MDCS) (1993-2019) and the Health Professionals Follow-Up Study (HPFS) (1996-2019) at Start of Follow-Up

Characteristic	Participants, No. (%)	
	MDCS (n = 10 270)	HPFS (n = 9337)
Age, median (IQR), y	59.0 (53.0-64.7)	65.1 (58.0-71.8)
Absolute PRS value, mean (SD)	24.4 (0.7)	24.3 (0.7)
PRS category		
0%-50%	5135 (50)	4669 (50)
50%-100%	5135 (50)	4668 (50)
Family history of any cancer (MDCS) or of prostate or breast cancer (HPFS)		
No	5727 (56)	6978 (75)
Yes	4543 (44)	2359 (25)
Combined genetic risk		
Lower: PRS 0%-50% and no family history	2857 (28)	3564 (38)
Higher: PRS 50%-100% or family history	7413 (72)	5773 (62)
Lifestyle score		
Healthy (3-6)	7222 (70)	6645 (71)
Unhealthy (0-2)	3048 (30)	2692 (29)

Abbreviation: PRS, polygenic risk score.

During a median (IQR) follow-up of 24 (16-26) years in the MDCS and 23 (14-25) years in the HPFS, 444 prostate cancer deaths were observed, of which 107 occurred by age 75 years and 337 after age 75 years. Both genetic and lifestyle factors were associated with higher rates of prostate cancer death, with a consistent pattern of greater associations for early prostate cancer death (Table 2). Compared with men at lower genetic risk, men at higher genetic risk had a 3-fold increased rate of early (HR, 3.26; 95% CI, 1.82-5.84) and a 2-fold increased rate of late (HR, 2.26; 95% CI, 1.70-3.01) prostate cancer death. In models stratified by genetic risk, an unhealthy lifestyle could only be shown to increase the rate of prostate cancer death among men at higher genetic risk (Table 3). Based on the detailed lifestyle categorization, both smoking and a BMI of 30 or greater were associated with an increased rate. Cohort-specific estimates were overall similar (eTable 3 and eTable 4 in Supplement 1).

**Table 2.** Pooled Hazard Ratios (HR) and 95% CIs for the Association Between Genetic Factors and the Lifestyle Score With All, Early (Up Until Age 75 Years), and Late (After Age 75 Years) Prostate Cancer (PCa) Deaths

Genetic or lifestyle group	All PCa deaths		Early PCa deaths		Late PCa deaths	
	Events/PY	HR (95% CI)^a^	Events/PY	HR (95% CI)^a^	Events/PY	HR (95% CI)^a^
PRS^b^						
0%-50%	122/313 414	1 [Reference]	26/194 533	1 [Reference]	96/118 837	1 [Reference]
50%-100%	322/296 471	2.87 (2.32-3.54)	81/188 659	3.19 (2.03-5.01)	241/107 792	2.77 (2.18-3.51)
Family history of cancer^b^						
No	241/422 111	1 [Reference]	47/263 167	1 [Reference]	194/158 900	1 [Reference]
Yes	203/187 775	1.33 (1.10-1.61)	60/120 026	2.10 (1.42-3.10)	143/67 728	1.15 (0.92-1.43)
Combined genetic risk^c^						
Lower: PRS 0%-50% and no family history	71/221560	1 [Reference]	13/137 090	1 [Reference]	58/84 441	1 [Reference]
Higher: PRS 50%-100% or family history	373/388 326	2.48 (1.92-3.20)	94/246 101	3.26 (1.82-5.84)	279/142 188	2.26 (1.70-3.01)
Lifestyle score^c^						
Healthy (3-6)	301/442 462	1 [Reference]	54/271 808	1 [Reference]	247/170 595	1 [Reference]
Unhealthy (0-2)	143/167 424	1.59 (1.30-1.95)	53/111 384	2.57 (1.75-3.77)	90/56 034	1.33 (1.04-1.70)

Abbreviations: PRS, polygenic risk score; PY, person-years.

^a^ HRs are adjusted for calendar year of inclusion, education (Malmö Diet and Cancer Study only), prostate-specific antigen screening history (Health Professionals Follow-Up Study [HPFS] only), principal components 1 to 3 of genetic variation (HPFS only), history of other cancers, history of diabetes, aspirin use, statin use, and total energy intake. Each genetic or lifestyle factor is also adjusted for the other presented factors. Cohort-specific hazard ratios are presented in eTable 3 in Supplement 1.

^b^ Results for PRS and family history of cancer are from the same model.

^c^ Results of combined genetic risk and lifestyle score are from the same model.

**Table 3.** Pooled Hazard Ratios (HR) and 95% CIs for the Association Between the Lifestyle Score and the Detailed Lifestyle Categorization With Prostate Cancer (PCa) Death According to Genetic Risk

Lifestyle group	All men, all PCa deaths		Men at lower genetic risk, all PCa deaths		Men at higher genetic risk, all PCa deaths	
	Events/PY	HR (95% CI)^a^	Events/PY	HR (95% CI)^a^	Events/PY	HR (95% CI)^a^
Lifestyle score						
Healthy (3-6)	301/442 462	1 [Reference]	52/160 805	1 [Reference]	249/281 658	1 [Reference]
Unhealthy (0-2)	143/167 424	1.59 (1.30-1.95)	19/60 755	1.16 (0.69-1.98)	124/106 669	1.69 (1.36-2.11)
Detailed lifestyle						
Nonsmokers with BMI <30, healthy^b^^,^^c^	37/52 630	1 [Reference]	7/17 501	1 [Reference]	30/35 131	1 [Reference]
Nonsmokers with BMI <30, moderate healthy^c^^,^^d^	236/373 776	1.30 (0.91-1.85)	38/142 387	0.90 (0.39-2.06)	198/231 389	1.39 (0.94-2.06)
Nonsmokers with BMI ≥30^c^	53/53 192	2.04 (1.33-3.14)	7/18 702	1.48 (0.51-4.30)	46/34 490	2.17 (1.36-3.48)
Smokers	118/130 286	1.65 (1.13-2.39)	19/42 970	1.38 (0.58-3.31)	99/87 318	1.70 (1.13-2.57)

Abbreviations: BMI, body mass index (calculated as weight in kilograms divided by height in meters squared); PY, person-years.

^a^ HRs are adjusted for calendar year of inclusion, education (Malmö Diet and Cancer Study only), prostate-specific antigen screening history (Health Professionals Follow-Up Study [HPFS] only), principal components 1 to 3 of genetic variation (HPFS only), history of other cancers, history of diabetes, aspirin use, statin use, and total energy intake. The hazard ratios are further adjusted for or stratified by genetic risk. Cohort-specific hazard ratios are presented in eTable 4 in Supplement 1.

^b^ Healthy was defined as high physical activity and healthy diet (fulfilling ≥2 of the dietary components).

^c^ Body mass index is calculated as weight in kilograms divided by height in meters squared.

^d^ Moderate healthy was defined as low physical activity or unhealthy diet (fulfilling ≤1 of the dietary components).

In both cohorts, the lifetime risks were lowest for men at lower genetic risk (0.6%-1.3% for the HPFS and the MDCS, respectively), without a consistent pattern by lifestyle (Figure; eTable 5 in Supplement 1). The lifetime risks were considerably higher for the remaining men, which reached 2.3% to 3.1% for the combined higher genetic risk group and ranged from 1.8% to 2.9% for men in the PRS 50% to 75% category to 3.1% to 4.9% for men in the PRS 75% to 100% category for the HPFS and the MDCS, respectively. There was a consistent pattern of higher risk among men with an unhealthy lifestyle, and in both cohorts, the highest lifetime risk was observed for men in the PRS 75% to 100% category with an unhealthy lifestyle.

**Figure.**
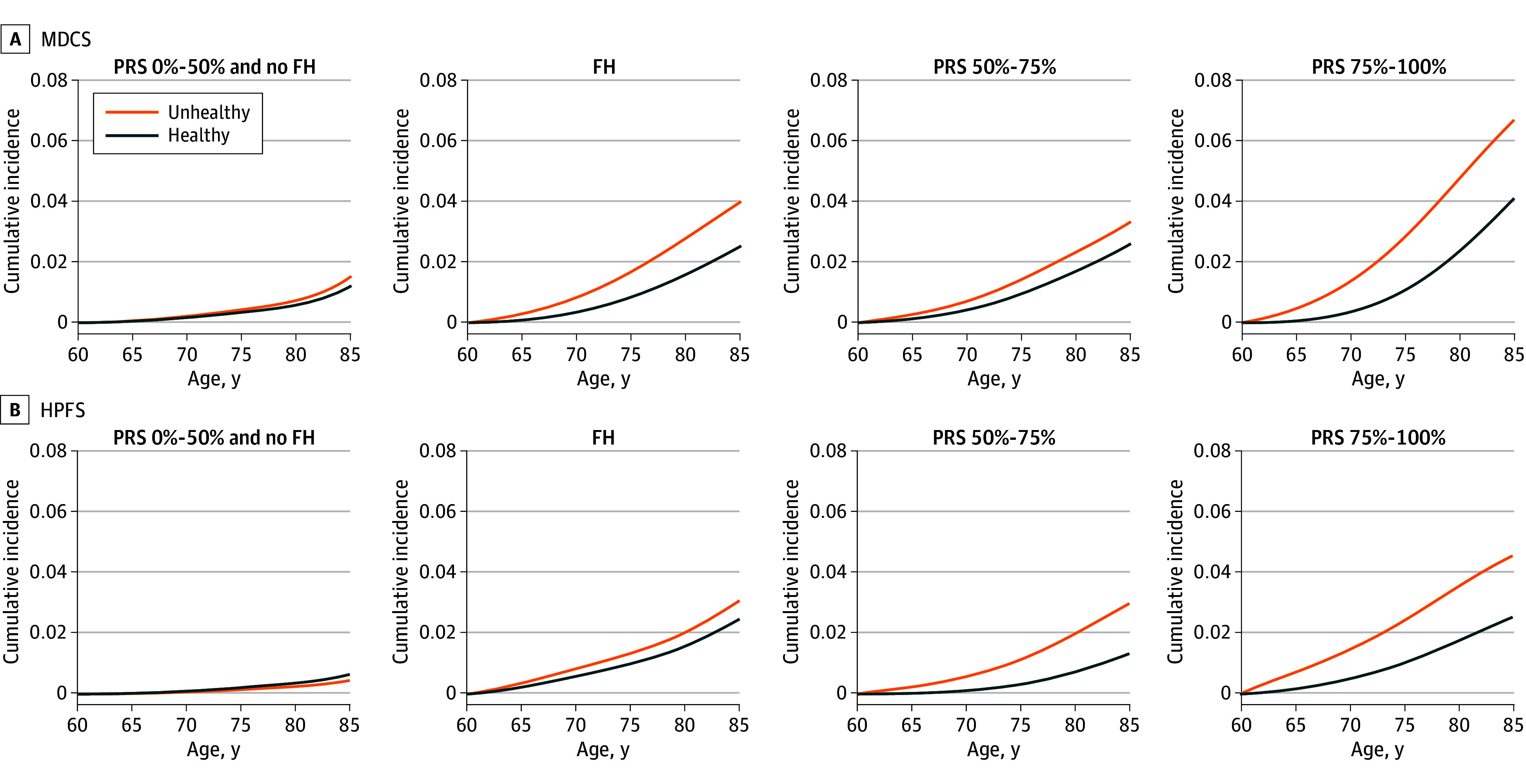
Absolute Risk (Cumulative Incidence) of Prostate Cancer Death Across the Age Span According to Different Genetic Risk Categories and the Lifestyle Score in the Malmö Diet and Cancer Study (MDCS) and the Health Professionals Follow-Up Study (HPFS) The 3 higher genetic risk categories may be potentially overlapping. Percentages are presented in a tabular format in eTable 5 in Supplement 1. FH indicates family history of cancer; PRS, polygenic risk score.

Most prostate cancer deaths occurred among men in the higher genetic risk group (Table 4 and eTable 6 in Supplement 1). Of the deaths occurring by age 75 years, 94 of 107 (88%) belonged to this group. Depending on the definition of a healthy lifestyle, we estimated that between 22% (95% CI, 11% to 34%) to 36% (95% CI, 12% to 60%) of all prostate cancer deaths occurring before the age of 75 years among men at higher genetic risk may be preventable (Table 4). In the sensitivity analysis using the alternative dietary factors, the corresponding estimate was 23% (95% CI, −9% to 54%) for the WCRF/AICR dietary factors and 39% (95% CI, 14% to 65%) for the combination high fiber, low processed meat, and low dairy product intake (eTable 7 in Supplement 1).

**Table 4.** Absolute Numbers and Pooled Estimated Percentage of Preventable Prostate Cancer (PCa) Deaths by Age 70 to Age 85 Years

Age, y	No. of PCa deaths (% of total PCa deaths)		% of Preventable PCa deaths (95% CI)^a^			
			If all men had a healthy lifestyle as defined by a lifestyle score of 3-6		If all men were nonsmokers with a BMI <30, and otherwise healthy^b^	
	All men	Higher genetic risk	All men	Higher genetic risk	All men	Higher genetic risk
70	49 (100)	43 (88)	19 (10-28)	28 (12-45)	38 (21-54)	44 (20-69)
75	107 (100)	94 (88)	14 (7.3-20)	22 (11-34)	31 (14-48)	36 (12-60)
80	202 (100)	180 (89)	10 (5.1-15)	18 (9.5-27)	26 (8.2-43)	30 (5.6-55)
85	303 (100)	263 (87)	7.4 (3.1-12)	15 (7.9-23)	22 (3.8-40)	26 (0.4-51)

Abbreviation: BMI, body mass index (calculated as weight in kilograms divided by height in meters squared).

^a^ Preventable deaths refer to deaths that would have been prevented had everyone been healthy at study entry. Healthy was defined both according to the lifestyle score and the detailed lifestyle categorization. Cohort-specific estimates are presented in eTable 6 in Supplement 1.

^b^ Healthy was defined as high physical activity and healthy diet (fulfilling ≥2 of the dietary components).

## Discussion

Based on data from 2 prospective cohort studies, this analysis provides evidence for targeting men at increased genetic risk with prevention strategies aimed at reducing premature deaths from prostate cancer. Men with a PRS above the median or a family history of cancer accounted for 88% of early prostate cancer deaths and had lifetime risks that were at least 2- to 3-fold higher compared with the remaining men. Importantly, we estimated that approximately one-third of early prostate cancer deaths among men in this group may be preventable through behaviors associated with a healthy lifestyle.

The extent to which men at high genetic risk benefit from targeted lifestyle or other interventions will become increasingly relevant with more widespread use of the PRS and other genetic testing. Except for a recommendation to start with earlier PSA screening among men with certain DNA repair gene alterations,^28^ there are currently no recommendations for men at high genetic risk. Lifestyle adaptions come with few if any harms and with several additional benefits to health, and strong promotions of healthy behaviors could be the first preventive action for men at increased genetic risk who also may be particularly motivated to adopt a healthy lifestyle. Such actions could go beyond the general advice of a healthy lifestyle that all men should be given, and may in the future include a personalized lifestyle, diet, or medication-based strategy. While the optimal lifestyle and diet for prevention has yet to be determined, it likely involves factors such as nonsmoking, maintaining a healthy weight, regular physical activity, and a healthy diet rich in vegetables and fiber and low in animal-based products.

This study supports previous studies in suggesting that a PRS for overall prostate cancer can be used to provide a robust risk stratification of prostate cancer mortality.^5,6^ Although PSA levels may provide good short-term risk stratification,^29,30^ the advantage of the PRS is that it can be measured before any cancer starts to develop. Ideally, the 2 should be combined.^31^ Our analysis suggests that the low-risk group may be the easiest to identify; the PRS, together with family history of cancer, appears to identify 30% to 40% of men in the population with a low risk of dying from prostate cancer (lifetime risk of 1% or less). Further work is required to refine the broad high genetic risk group. We previously showed that men with a family history of prostate or breast cancer who were in the PRS 75% to 100% category had the highest lifetime risk, but the risk was nearly as high for men with a family history in the PRS 50% to 75% category.^7^ A genetic risk-based screening program would be more powerful if we could identify the 20% of the population in which 90% of all prostate cancer deaths occurred.^32^

Nevertheless, the available PRS already provides strong risk stratification for both early and late prostate cancer mortality as demonstrated in the current analysis, and may be informative for clinical practice. Men in the higher genetic risk group may benefit from earlier screening; previous work has shown that men at the highest genetic risk reach the same level of absolute risk 15 to 16 years earlier than the average man^7,24^ and our current work suggests that the risk can be reduced through modifiable factors, which should motivate preventive actions in this group.

The 2 cohort studies included in this analysis were similar in both design and follow-up, but some differences should be noted. PSA testing was not available at study entry for men in the MDCS and in Sweden in general,^33^ whereas the uptake of PSA testing was quicker in the US and among men in the HPFS in particular.^34^ While the MDCS is population-based, the HPFS includes health professionals who generally have more favorable lifestyle characteristics compared with the broader US population.^35^ A higher proportion of men with unhealthy lifestyles may increase the percentage of preventable deaths, as illustrated in a previous study^35^ estimating that adherence to a healthy lifestyle could have prevented 21% of overall prostate cancer deaths in the full HPFS cohort, and 40% of prostate cancer deaths in the broader US population. Factors indirectly associated with a healthy lifestyle, such as earlier detection and treatment, are likely better controlled for in the HPFS.

### Limitations and Strengths

This study should be viewed considering its limitations. Since it is possible that differences in prostate cancer testing and treatment account for some (but most likely not all)^6,14,36^ of the observed association between a healthy lifestyle and prostate cancer death, this analysis should be viewed as providing an estimate of what is achievable in terms of prevention had everyone had a healthy lifestyle and adopted behaviors that go along with such a lifestyle. It is possible that our estimates include deaths preventable by equal opportunities for early detection and treatment—this does not change the overall conclusion but indicates that the exact mechanisms are difficult to determine. A randomized clinical trial could provide a more defined estimate, but such a study may not always be feasible to perform because it would likely require a long adherence to the intervention (if it would be possible to randomize) and a long follow-up period. One other limitation is that we only considered factors at study entry. While this has advantages (such as limiting the influence of reverse causation), it does not reflect later changes and could potentially underestimate the percentage of preventable deaths.^37^ This analysis was further limited to men of European ancestry, but similar associations have been observed for men in the more diverse Million Veteran Program.^38^ Our study has numerous strengths, of which the most important is the inclusion of 2 independent and large cohorts of men with 2 decades of follow-up. Although the populations represented by the 2 cohorts were quite different, the cohort-specific results were similar, suggesting that our findings are generalizable to a wider population.

## Conclusions

In conclusion, this study suggests that men at increased genetic risk should be targeted for prostate cancer prevention strategies. Approximately one-third of premature prostate cancer deaths in this group may be preventable by healthy lifestyle choices or alternatively, if the findings are explained by earlier detection and treatment, by equal opportunities to receive optimal health care. Implementing interventions among men at increased genetic risk may substantially reduce the number of early deaths due to prostate cancer.

## Notes

Supplement 1. **eTable 1.** The Nondietary and Dietary Components of the Healthy Lifestyle Score, Including Alternative Versions **eTable 2.** Cohort-Specific Hazard Ratios and 95% CIs for the Association Between the Different Polygenic Risk Scores With All, Early (Up Until Age 75 Years) and Late (After Age 75 Years) Prostate Cancer Deaths **eTable 3.** Cohort-Specific Hazard Ratios and 95% CIs for the Association Between Genetic Factors and Lifestyle Score With All, Early (Up Until Age 75 Years) and Late (After Age 75 Years) Prostate Cancer Deaths **eTable 4.** Cohort-Specific Hazard Ratios and 95% CIs for the Association Between the Lifestyle Score and the Detailed Lifestyle Categorization With Prostate Cancer Death According to Genetic Risk **eTable 5.** Cohort-Specific Absolute Risks (Cumulative Incidence) of Prostate Cancer Death by Ages 70, 75, 80 and 85 Years According to Genetic Risk and Lifestyle **eTable 6.** Cohort-Specific Absolute Numbers and Estimated Percentage of Preventable Prostate Cancer Deaths by Age 70 to Age 85 Years **eTable 7.** Sensitivity Analysis of the Estimated Percentage of Preventable Prostate Cancer Deaths by Age 70 to Age 85 Years Based on Different Definitions of a Healthy Diet **eAppendix 1. **Supplementary Contributor Information **eAppendix 2. **Supplementary Acknowledgements

Supplement 2.
Data Sharing Statement
