# Supplementary material for: Early Prostate Cancer Deaths Among Men With Higher vs Lower Genetic Risk
Source: JAMA Netw Open. 2024 Jul 3;7(7):e2420034. doi: 10.1001/jamanetworkopen.2024.20034 (PMC11222990; doi:10.1001/jamanetworkopen.2024.20034)
Supplement: Supplement 1. — eTable 1. The Nondietary and Dietary Components of the Healthy Lifestyle Score, Including Alternative Versions eTable 2. Cohort-Specific Hazard Ratios and 95% CIs for the Association Between the Different Polygenic Risk Scores With All, Early (Up Until Age 75 Years) and Late (After Age 75 Years) Prostate Cancer Deaths eTable 3. Cohort-Specific Hazard Ratios and 95% CIs for the Association Between Genetic Factors and Lifestyle Score With All, Early (Up Until Age 75 Years) and Late (After Age 75 Years) Prostate Cancer Deaths eTable 4. Cohort-Specific Hazard Ratios and 95% CIs for the Association Between the Lifestyle Score and the Detailed Lifestyle Categorization With Prostate Cancer Death According to Genetic Risk eTable 5. Cohort-Specific Absolute Risks (Cumulative Incidence) of Prostate Cancer Death by Ages 70, 75, 80 and 85 Years According to Genetic Risk and Lifestyle eTable 6. Cohort-Specific Absolute Numbers and Estimated Percentage of Preventable Prostate Cancer Deaths by Age 70 to Age 85 Years eTable 7. Sensitivity Analysis of the Estimated Percentage of Preventable Prostate Cancer Deaths by Age 70 to Age 85 Years Based on Different Definitions of a Healthy Diet eAppendix 1. Supplementary Contributor Information eAppendix 2. Supplementary Acknowledgements [file jamanetwopen-e2420034-s001.pdf]

Supplemental Online Content

Plym A, Zhang Y, Stopsack KH, et al. Early prostate cancer deaths among men with higher vs lower genetic risk. *JAMA Netw Open*. 2024;7(7):e2420034. doi:10.1001/jamanetworkopen.2024.20034

eTable 1. The Nondietary and Dietary Components of the Healthy Lifestyle Score, Including Alternative Versions

eTable 2. Cohort-Specific Hazard Ratios and 95% CIs for the Association Between the Different Polygenic Risk Scores With All, Early (Up Until Age 75 Years) and Late (After Age 75 Years) Prostate Cancer Deaths

eTable 3. Cohort-Specific Hazard Ratios and 95% CIs for the Association Between Genetic Factors and Lifestyle Score With All, Early (Up Until Age 75 Years) and Late (After Age 75 Years) Prostate Cancer Deaths

eTable 4. Cohort-Specific Hazard Ratios and 95% CIs for the Association Between the Lifestyle Score and the Detailed Lifestyle Categorization With Prostate Cancer Death According to Genetic Risk

eTable 5. Cohort-Specific Absolute Risks (Cumulative Incidence) of Prostate Cancer Death by Ages 70, 75, 80 and 85 Years According to Genetic Risk and Lifestyle

eTable 6. Cohort-Specific Absolute Numbers and Estimated Percentage of Preventable Prostate Cancer Deaths by Age 70 to Age 85 Years

eTable 7. Sensitivity Analysis of the Estimated Percentage of Preventable Prostate Cancer Deaths by Age 70 to Age 85 Years Based on Different Definitions of a Healthy Diet

eAppendix 1. Supplementary Contributor Information

eAppendix 2. Supplementary Acknowledgements

This supplemental material has been provided by the authors to give readers additional information about their work.

© 2024 Plym A et al. *JAMA Network Open.*

eTable 1. The Nondietary and Dietary Components of the Healthy Lifestyle Score, Including Alternative Versions. One point was assigned for each factor fulfilled.

		MDCS	HPFS
Non-dietary factors [a]	Not smoking	Never smoker or quit ≥ 10yrs	
	Healthy weight	Body mass index < 30 kg/m^2	
	High physical activity	Physical activity score quartile 3–quartile 5	≥3 h/week vigorous activity and/or ≥7 h/week brisk walking
Dietary factors [a]	High intake of tomatoes	≥2 or more servings/week of raw tomatoes	≥7 servings/week of raw tomatoes, tomato juice, tomato sauce, salsa, pizza
	High intake of fatty fish	≥1 servings/week of fatty fish (>5% fat)	≥1 serving/week mackerel, salmon, sardines, bluefish, swordfish
	Low intake of processed meat	<3 servings/week	
Alternative dietary factors from WCRF/AICR [b]	Low or moderate intake of alcohol	≤28 g/day	
	High fruit and vegetable intake	≥400 g/day	
	High fiber intake	≥30 g/day	
	Low intake of processed and red meat	<100 g/week of processed meat and <500 g/week of red meat	
	Low intake of sugar-sweetened beverages	≤250 g/day	
	Low intake of energy-dense food	<2 servings/day	Tertile 1
Alternative dietary factors based on the 2022 literature review [c]	High fiber intake	≥30 g/day	
	Low intake of processed meat	<3 servings/week	
	Low intake of dairy products	<2.5 servings/day	

Abbreviations: HPFS, Health Professionals Follow-up Study; MDCS, Malmö Diet and Cancer Study.
a. In the HPFS, all definitions were the same as in the publication by Kenfield *et al.* (PMID 26577654). In the MDCS, the definition of a high physical activity was based on the publication by Li *et al.* (PMID 19639000) and serving sizes were adapted from Dimovski *et al.* (PMID 31775698).
b. In the HPFS, the dietary items were the same as in the publication by Graff *et al.* (PMID 36028533). In the MDCS, the dietary items were the same as in the publication by Drake *et al.* (PMID 31077359).
c. Based on the 2022 literature review by Bergengren *et al.* (PMID: 37202314). The cut-points for fiber and processed meat were borrowed from the above scores. The cut-point for dairy products was based on the publication by Dimovski *et al*. (PMID 31775698). Dairy products includes both high and low-fat milk, cheese, and yoghurt.

© 2024 Plym A et al. *JAMA Network Open.*

eTable 2. Cohort-Specific Hazard Ratios and 95% CIs for the Association Between the Different Polygenic Risk Scores With All, Early (Up Until Age 75 Years) and Late (After Age 75 Years) Prostate Cancer Deaths.

	All PCa deaths		Early PCa deaths		Late PCa deaths	
	Events/PY	HR (95% CI) [a]	Events/PY	HR (95% CI) [a]	Events/PY	HR (95% CI) [a]
MDCS						
**400-variant PRS**						
Per s.d.	288/211370	1.84 (1.65-2.06)	75/150086	1.86 (1.50-2.30)	213/61284	1.83 (1.61-2.09)
Quartiles						
0-25%	33/53286	1 (Ref.)	7/37747	1 (Ref.)	26/15538	1 (Ref.)
25-50%	41/53475	1.23 (0.78-1.95)	10/37649	1.44 (0.55-3.79)	31/15825	1.17 (0.70-1.98)
50-75%	79/52296	2.54 (1.69-3.81)	24/37320	3.50 (1.51-8.13)	55/14976	2.28 (1.43-3.64)
75-100%	135/52314	4.33 (2.95-6.33)	34/37370	5.01 (2.22-11.32)	101/14945	4.13 (2.68-6.36)
**451-variant PRS**						
Per s.d.	288/211370	1.93 (1.72-2.17)	75/150086	1.86 (1.50-2.31)	213/61284	1.96 (1.71-2.25)
Quartiles						
0-25%	34/53719	1 (Ref.)	8/38033	1 (Ref.)	26/15686	1 (Ref.)
25-50%	37/53032	1.07 (0.67-1.70)	10/37536	1.28 (0.50-3.23)	27/15495	1.01 (0.59-1.73)
50-75%	76/52642	2.30 (1.53-3.44)	22/37215	2.85 (1.27-6.41)	54/15426	2.15 (1.34-3.43)
75-100%	141/51977	4.53 (3.11-6.59)	35/37301	4.60 (2.13-9.93)	106/14676	4.51 (2.93-6.93)

© 2024 Plym A et al. *JAMA Network Open.*

eTable 2 cont.

	All PCa deaths		Early PCa deaths		Late PCa deaths	
	Events/PY	HR (95% CI) [a]	Events/PY	HR (95% CI) [a]	Events/PY	HR (95% CI) [a]
HPFS						
**400-variant PRS**						
Per s.d.	156/398516	1.71 (1.45-2.02)	32/233106	1.60 (1.20-2.14)	124/165345	1.73 (1.42-2.11)
Quartiles						
0-25%	20/106609	1 (Ref.)	2/60310	1 (Ref.)	18/46279	1 (Ref.)
25-50%	28/100045	1.47 (0.83-2.60)	7/58827	3.58 (0.73-17.46)	21/41194	1.24 (0.66-2.33)
50-75%	43/100781	2.34 (1.37-3.99)	11/59871	5.58 (1.19-26.19)	32/40897	1.97 (1.10-3.52)
75-100%	65/91081	3.99 (2.40-6.62)	12/54098	6.35 (1.36-29.59)	53/36975	3.73 (2.17-6.41)
**451-variant PRS**						
Per s.d.	156/398516	1.69 (1.45-1.98)	32/233106	1.57 (1.12-2.19)	124/165345	1.72 (1.44-2.06)
Quartiles						
0-25%	20/108014	1 (Ref.)	4/61075	1 (Ref.)	16/46909	1 (Ref.)
25-50%	22/101874	1.14 (0.62-2.09)	4/59360	1.11 (0.28-4.40)	18/42493	1.17 (0.60-2.31)
50-75%	50/100491	2.79 (1.66-4.70)	10/58834	2.60 (0.81-8.35)	40/41648	2.82 (1.57-5.05)
75-100%	64/88136	3.90 (2.35-6.47)	14/53837	3.83 (1.22-12.01)	50/34295	3.98 (2.26-7.01)

Abbreviations: HPFS, Health Professionals Follow-up Study; MDCS, Malmö Diet and Cancer Study; PCa, prostate cancer; PRS, polygenic risk score; PY, person-years; s.d., standard deviation.

a. Hazard ratios are adjusted for calendar year of inclusion, education (MDCS only), PSA screening history (HPFS only), principal components (PCs) 1-3 of genetic variation (HPFS only), history of other cancers, history of diabetes, aspirin use, statin use, total energy intake, and the lifestyle score.

© 2024 Plym A et al. *JAMA Network Open.*

eTable 3. Cohort-Specific Hazard Ratios and 95% CIs for the Association Between Genetic Factors and Lifestyle Score With All, Early (Up Until Age 75 Years) and Late (After Age 75 Years) Prostate Cancer Deaths.

	All PCa deaths		Early PCa deaths		Late PCa deaths	
	Events/PY	HR (95% CI) [a]	Events/PY	HR (95% CI) [a]	Events/PY	HR (95% CI) [a]
**PRS** [b]						
MDCS						
0-50%	74/106760	1 (Ref.)	17/75397	1 (Ref.)	57/31364	1 (Ref.)
50-100%	214/104610	3.08 (2.36-4.01)	58/74690	3.49 (2.03-5.99)	156/29920	2.95 (2.18-4.00)
HPFS						
0-50%	48/206654	1 (Ref.)	9/119136	1 (Ref.)	39/87473	1 (Ref.)
50-100%	108/191861	2.53 (1.79-3.58)	23/113969	2.61 (1.16-5.90)	85/77872	2.50 (1.70-3.67)
**Family history of cancer** [b]						
MDCS						
No	139/119169	1 (Ref.)	32/86230	1 (Ref.)	107/32939	1 (Ref.)
Yes	149/92201	1.26 (1.00-1.58)	43/63857	1.75 (1.10-2.76)	106/28345	1.12 (0.86-1.47)
HPFS						
No	102/302942	1 (Ref.)	15/176937	1 (Ref.)	87/125961	1 (Ref.)
Yes	54/95574	1.51 (1.08-2.11)	17/56169	3.35 (1.61-6.96)	37/39383	1.20 (0.81-1.76)
**Combined genetic risk** [c]						
MDCS						
Lower	40/59881	1 (Ref.)	10/43305	1 (Ref.)	30/16576	1 (Ref.)
Higher	248/151489	2.33 (1.67-3.25)	65/106781	2.63 (1.35-5.11)	183/44708	2.23 (1.52-3.28)
HPFS						
Lower	31/161679	1 (Ref.)	3/93785	1 (Ref.)	28/67865	1 (Ref.)
Higher	125/236837	2.71 (1.82-4.02)	29/139320	6.55 (1.99-21.63)	96/97480	2.30 (1.50-3.52)

© 2024 Plym A et al. *JAMA Network Open.*

eTable 3 cont.

	All PCa deaths		Early PCa deaths		Late PCa deaths	
	Events/PY	HR (95% CI) [a]	Events/PY	HR (95% CI) [a]	Events/PY	HR (95% CI) [a]
**Lifestyle score** [c]						
MDCS						
Healthy	195/151947	1 (Ref.)	37/104590	1 (Ref.)	158/47357	1 (Ref.)
Unhealthy	93/59423	1.69 (1.31-2.17)	38/45496	2.65 (1.68-4.20)	55/13927	1.40 (1.02-1.90)
HPFS						
Healthy	106/290515	1 (Ref.)	17/167218	1 (Ref.)	89/123238	1 (Ref.)
Unhealthy	50/108001	1.42 (1.01-2.00)	15/65888	2.39 (1.20-4.78)	35/42107	1.24 (0.83-1.84)

Abbreviations: HPFS, Health Professionals Follow-up Study; MDCS, Malmö Diet and Cancer Study; PCa, prostate cancer; PRS, polygenic risk score; PY, person-years.
a. Hazard ratios are adjusted for calendar year of inclusion, education (MDCS only), PSA screening history (HPFS only), principal components (PCs) 1-3 of genetic variation (HPFS only), history of other cancers, history of diabetes, aspirin use, statin use, and total energy intake. Each genetic/lifestyle factor is also adjusted for the other presented factors.
b-c. The presented results denoted with the same letter are from the same model.

© 2024 Plym A et al. *JAMA Network Open.*

eTable 4. Cohort-Specific Hazard Ratios and 95% CIs for the Association Between the Lifestyle Score and the Detailed Lifestyle Categorization With Prostate Cancer Death According to Genetic Risk.

	All men		Men at lower genetic risk		Men at higher genetic risk	
	All PCa deaths		All PCa deaths		All PCa deaths	
	Events/PY	HR (95% CI) [a]	Events/PY	HR (95% CI) [a]	Events/PY	HR (95% CI) [a]
**Lifestyle score**						
MDCS						
Healthy	195/151947	1 (Ref.)	27/42367	1 (Ref.)	168/109581	1 (Ref.)
Unhealthy	93/59423	1.69 (1.31-2.17)	13/17514	1.52 (0.79-2.96)	80/41909	1.72 (1.31-2.25)
HPFS						
Healthy	106/290515	1 (Ref.)	25/118438	1 (Ref.)	81/172077	1 (Ref.)
Unhealthy	50/108001	1.42 (1.01-2.00)	6/43241	0.72 (0.30-1.75)	44/64760	1.64 (1.12-2.39)
**Detailed lifestyle**						
MDCS						
Non-smokers, BMI <30 kg/m^2, healthy [b]	30/24778	1 (Ref.)	5/6941	1 (Ref.)	25/17838	1 (Ref.)
Non-smokers, BMI <30 kg/m^2, moderate healthy [c]	128/88501	1.31 (0.88-1.95)	16/24496	0.91 (0.33-2.49)	112/64005	1.39 (0.90-2.14)
Non-smokers, BMI ≥30 kg/m^2	35/16216	2.04 (1.25-3.34)	4/4620	1.58 (0.42-5.91)	31/11596	2.13 (1.25-3.62)
Smokers	95/81874	1.52 (1.00-2.30)	15/23824	1.33 (0.48-3.68)	80/58051	1.55 (0.99-2.44)
HPFS						
Non-smokers, BMI <30 kg/m^2, healthy [b]	7/27852	1 (Ref.)	2/10560	1 (Ref.)	5/17293	1 (Ref.)
Non-smokers, BMI <30 kg/m^2, moderate healthy [c]	108/285275	1.27 (0.59-2.74)	22/117891	0.88 (0.21-3.79)	86/167384	1.43 (0.58-3.53)
Non-smokers, BMI ≥30 kg/m^2	18/36976	2.05 (0.84-4.99)	3/14082	1.30 (0.21-7.99)	15/22894	2.34 (0.84-6.54)
Smokers	23/48412	2.35 (0.99-5.55)	4/19146	1.53 (0.28-8.46)	19/29267	2.67 (0.98-7.25)

Abbreviations: BMI, body mass index; HPFS, Health Professionals Follow-up Study; MDCS, Malmö Diet and Cancer Study; PCa, prostate cancer; PY, person-years; Ref., reference.
a. Hazard ratios are adjusted for calendar year of inclusion, education (MDCS only), PSA screening history (HPFS only), principal components (PCs) 1-3 of genetic variation (HPFS only), history of other cancers, history of diabetes, aspirin use, statin use, and total energy intake.

© 2024 Plym A et al. *JAMA Network Open.*

eTable 4. cont.

b. Healthy was defined as high physical activity and healthy diet (fulfilling ≥2 of the dietary components).
c. Moderate healthy was defined as low physical activity or unhealthy diet (fulfilling ≤1 of the dietary components).

© 2024 Plym A et al. *JAMA Network Open.*

eTable 5. Cohort-Specific Absolute Risks (Cumulative Incidence) of Prostate Cancer Death by Ages 70, 75, 80 and 85 Years According to Genetic Risk and Lifestyle.

		Lifestyle score	
	Population average	Healthy (3-5)	Unhealthy (0-2)
Age, years	% (95% CI)	% (95% CI)	% (95% CI)
MDCS			
All men			
70	0.4 (0.3-0.5)	0.3 (0.2-0.4)	0.6 (0.4-1.0)
75	0.9 (0.7-1.1)	0.7 (0.5-0.9)	1.2 (0.9-1.7)
80	1.6 (1.4-1.9)	1.3 (1.1-1.6)	2.1 (1.7-2.7)
85	2.6 (2.3-3.0)	2.3 (1.9-2.7)	3.3 (2.6-4.1)
Lower genetic risk: PRS 0-50% and no family history			
70	0.2 (0.1-0.5)	0.2 (0.1-0.4)	0.2 (0.1-0.6)
75	0.4 (0.2-0.7)	0.3 (0.2-0.7)	0.4 (0.2-0.9)
80	0.6 (0.4-1.0)	0.6 (0.3-1.0)	0.7 (0.4-1.4)
85	1.3 (0.9-1.9)	1.2 (0.8-1.9)	1.5 (0.9-2.7)
Higher genetic risk: PRS 50-100% or family history			
70	0.5 (0.3-0.7)	0.3 (0.2-0.5)	0.7 (0.5-1.2)
75	1.1 (0.9-1.3)	0.8 (0.6-1.1)	1.5 (1.1-2.2)
80	2.0 (1.7-2.4)	1.6 (1.3-2.0)	2.6 (2.0-3.5)
85	3.1 (2.7-3.6)	2.7 (2.2-3.2)	3.9 (3.1-4.9)
Family history			
70	0.5 (0.3-0.8)	0.3 (0.2-0.6)	0.8 (0.4-1.6)
75	1.1 (0.8-1.5)	0.8 (0.6-1.2)	1.7 (1.1-2.6)
80	2.0 (1.6-2.5)	1.6 (1.2-2.1)	2.8 (2.0-3.9)
85	3.1 (2.6-3.7)	2.5 (2.0-3.2)	4.0 (3.0-5.3)
PRS 50-75%			
70	0.5 (0.3-0.9)	0.4 (0.2-0.9)	0.7 (0.3-1.7)
75	1.1 (0.8-1.6)	1.0 (0.6-1.5)	1.4 (0.8-2.6)
80	1.9 (1.5-2.6)	1.7 (1.2-2.4)	2.3 (1.4-3.8)
85	2.9 (2.3-3.8)	2.6 (1.9-3.6)	3.3 (2.2-5.1)
PRS 75-100%			
70	0.6 (0.4-1.1)	0.3 (0.2-0.7)	1.4 (0.7-2.6)
75	1.6 (1.2-2.1)	1.1 (0.7-1.6)	2.8 (1.8-4.4)
80	3.1 (2.5-3.9)	2.4 (1.8-3.2)	4.8 (3.4-6.8)
85	4.9 (4.0-5.9)	4.1 (3.2-5.3)	6.7 (5.0-9.1)

© 2024 Plym A et al. *JAMA Network Open.*

eTable 5 cont.

		Lifestyle score	
	Population average	Healthy (3-5)	Unhealthy (0-2)
Age, years	% (95% CI)	% (95% CI)	% (95% CI)
HPFS			
All men [a]			
70	0.4 (0.3-0.5)	0.3 (0.3-0.4)	0.5 (0.4-0.7)
75	0.7 (0.7-0.8)	0.7 (0.6-0.8)	0.9 (0.8-1.1)
80	1.2 (1.1-1.3)	1.1 (1.0-1.3)	1.4 (1.2-1.7)
85	1.8 (1.7-2.0)	1.7 (1.6-1.9)	2.0 (1.8-2.3)
Lower genetic risk: PRS 0-50% and no family history			
70	0.1 (0.0-0.5)	0.1 (0.0-0.5)	0.1 (0.0-0.4)
75	0.2 (0.1-0.6)	0.2 (0.1-0.7)	0.1 (0.0-0.5)
80	0.3 (0.2-0.7)	0.4 (0.2-0.8)	0.3 (0.1-0.7)
85	0.6 (0.3-1.0)	0.7 (0.4-1.2)	0.5 (0.2-1.1)
Higher genetic risk: PRS 50-100% or family history			
70	0.5 (0.3-0.8)	0.3 (0.2-0.7)	0.8 (0.4-1.7)
75	0.9 (0.6-1.4)	0.7 (0.5-1.1)	1.5 (0.9-2.5)
80	1.5 (1.2-2.0)	1.3 (0.9-1.7)	2.3 (1.5-3.4)
85	2.3 (1.9-2.9)	2.0 (1.5-2.6)	3.2 (2.3-4.5)
Family history			
70	0.7 (0.3-1.3)	0.6 (0.3-1.3)	0.8 (0.3-2.2)
75	1.1 (0.7-1.8)	1.0 (0.6-1.8)	1.3 (0.6-2.9)
80	1.7 (1.2-2.5)	1.6 (1.0-2.5)	2.0 (1.1-3.8)
85	2.7 (1.9-3.7)	2.5 (1.7-3.7)	3.1 (1.8-5.2)
PRS 50-75%			
70	0.2 (0.1-0.7)	0.1 (0.0-0.5)	0.6 (0.2-2.0)
75	0.6 (0.3-1.1)	0.3 (0.1-0.8)	1.1 (0.5-2.7)
80	1.1 (0.7-1.7)	0.7 (0.4-1.4)	2.0 (1.0-3.8)
85	1.8 (1.2-2.7)	1.3 (0.8-2.3)	3.0 (1.8-5.1)
PRS 75-100%			
70	0.8 (0.3-1.7)	0.5 (0.2-1.3)	1.5 (0.5-4.0)
75	1.4 (0.8-2.4)	1.0 (0.5-2.0)	2.4 (1.1-5.2)
80	2.2 (1.5-3.3)	1.8 (1.1-2.8)	3.6 (2.0-6.4)
85	3.1 (2.3-4.1)	2.5 (1.8-3.7)	4.6 (2.8-7.4)

Abbreviations: BMI, body mass index; HPFS, Health Professionals Follow-up Study; MDCS, Malmö Diet and Cancer Study.
a. Absolute risks for all men in HPFS was calculated based on 44,077 men with complete lifestyle data from the underlying cohort.

© 2024 Plym A et al. *JAMA Network Open.*

eTable 6. Cohort-Specific Absolute Numbers and Estimated Percentage of Preventable Prostate Cancer Deaths by Age 70 to Age 85 Years.

Preventable deaths refer to deaths that would have been prevented had everyone been healthy at study entry. Healthy was defined both according to the lifestyle score and the detailed lifestyle categorization.

	No. of PCa deaths/No. of total PCa deaths (%)		Percentage of preventable PCa deaths			
			If all men had a healthy lifestyle as defined by a lifestyle score of 3-6 (95% CI)		If all men were non-smokers with a BMI <30 kg/m^2, and otherwise healthy (95% CI) [a]	
Age, years	All men	Higher genetic risk	All men [b]	Higher genetic risk	All men [b]	Higher genetic risk
MDCS						
70	32 (100)	28 (88)	27 (7.0-46)	28 (8.0-48)	47 (22-72)	47 (20-73)
75	75 (100)	65 (87)	21 (7.0-34)	22 (8.4-36)	36 (11-61)	37 (11-64)
80	140 (100)	125 (89)	16 (6.2-26)	18 (7.9-29)	28 (2.0-54)	31 (4.0-58)
85	207 (100)	181 (87)	13 (4.7-21)	15 (6.5-24)	22 (-5.3-49)	26 (-2.1-54)
HPFS						
70	17 (100)	15 (88)	17 (7.0-27)	28 (-0.6-57)	30 (8.1-53)	31 (-30-92)
75	32 (100)	29 (91)	12 (4.3-19)	22 (1.1-44)	26 (3.4-49)	29 (-33-90)
80	62 (100)	55 (89)	8.1 (2.2-14)	18 (1.7-35)	24 (0.2-47)	27 (-35-90)
85	96 (100)	82 (85)	5.3 (0.3-10)	15 (1.4-29)	22 (-2.2-46)	26 (-37-89)

Abbreviations: BMI, body mass index; HPFS, Health Professionals Follow-up Study; MDCS, Malmö Diet and Cancer Study; PCa, prostate cancer.

a. Healthy was defined as high physical activity and healthy diet (fulfilling ≥2 of the dietary components).

b. The percentage of preventable deaths for all men in HPFS was calculated based on 44,077 men with complete lifestyle data from the underlying cohort of men.

© 2024 Plym A et al. *JAMA Network Open.*

eTable 7. Sensitivity Analysis of the Estimated Percentage of Preventable Prostate Cancer Deaths by Age 70 to Age 85 Years Based on Different Definitions of a Healthy Diet: fulfilling ≥4 of the dietary recommendations from the WCRF/AICR, or fulfilling ≥2 components identified in the 2022 literature review (high fiber, low processed meat, and low dairy product intake). Preventable deaths refer to deaths that would have been prevented had everyone been healthy at study entry according to the detailed lifestyle categorization.

	Percentage of preventable PCa deaths (95% CI)			
	If all men were non-smokers with a BMI <30 kg/m2, and otherwise healthy **based on the WCRF/AICR dietary factors** [a]		If all men were non-smokers with a BMI <30 kg/m2, and otherwise healthy **based on fiber, processed meat, and dairy product intake** [b]	
Age, years	All men [c]	Higher genetic risk	All men [c]	Higher genetic risk
Both cohorts (pooled)				
70	31 (13-49)	30 (-0.1-61)	37 (22-53)	46 (21-70)
75	24 (5.3-43)	23 (-8.7-54)	30 (14-46)	39 (14-65)
80	20 (0.5-39)	18 (-15-50)	25 (8.7-42)	35 (9.1-62)
85	17 (-3.1-37)	14 (-19-47)	22 (4.9-39)	33 (5.4-60)
MDCS				
70	41 (5.7-77)	36 (-4.2-76)	63 (37-90)	55 (23-88)
75	29 (-9.9-68)	25 (-18-67)	56 (26-85)	48 (12-83)
80	20 (-22-62)	17 (-28-62)	50 (17-83)	42 (4.3-80)
85	13 (-31-58)	11 (-36-58)	46 (10-81)	38 (-2.2-78)
HPFS				
70	27 (5.9-48)	23 (-24-70)	24 (5.1-43)	33 (-4.3-70)
75	23 (1.1-44)	20 (-26-67)	20 (0.5-39)	31 (-6.0-67)
80	20 (-2.1-42)	19 (-28-65)	17 (-2.7-36)	29 (-7.5-66)
85	18 (-4.6-40)	17 (-30-64)	15 (-5.1-34)	28 (-8.8-65)

Abbreviations: BMI, body mass index; HPFS, Health Professionals Follow-up Study; MDCS, Malmö Diet and Cancer Study; PCa, prostate cancer; WCRF/AICR, World Cancer Research Fund/American Institute of Cancer Research.

a. Healthy was defined as high physical activity and healthy diet (fulfilling ≥4 of the dietary recommendations from WCRF).

b. Healthy was defined as high physical activity and healthy diet (fulfilling ≥2 of the dietary components identified in the 2022 literature review).

c. The percentage of preventable deaths for all men in HPFS was calculated based on 44,077 men with complete lifestyle data from the underlying cohort.

© 2024 Plym A et al. *JAMA Network Open.*

eAppendix 1. Supplementary Contributor Information

Regeneron Genetics Center members

RGC Management & Leadership Team
Gonçalo Abecasis, D. Phil., Adolfo Ferrando, M.D., Ph.D., Aris Baras, M.D., Michael Cantor, M.D., Giovanni Coppola, M.D., Andrew Deubler, M.P.S., Aris Economides, Ph.D., Luca A Lotta, M.D., Ph.D., John D Overton, Ph.D., Jeffrey G Reid, Ph.D., Alan Shuldiner, M.D., Katherine Siminovitch, M.D.

Contribution: All authors contributed to securing funding, study design and oversight. All authors reviewed the final version of the manuscript.

Sequencing & Lab Operations
John D Overton, Ph.D., Christina Beechert, Erin D Brian, Laura M Cremona, Ph.D., Hang Du, Caitlin Forsythe, M.S., Zhenhua Gu, M.S., Kristy Guevara, M.S., Michael Lattari, Alexander Lopez, M.S., Kia Manoochehri, Prathyusha Challa, M.S., Manasi Pradhan, M.S., Raymond Reynoso, Ricardo Schiavo, Maria Sotiropoulos Padilla, M.S., Chenggu Wang, M.S., Sarah E Wolf, M.S.

Contribution: Performed and are responsible for sample genotyping and exome sequencing, conceived and are responsible for laboratory automation, and responsible for sample tracking and the library information management system.

Clinical Informatics
Michael Cantor M.D. , Amelia Averitt, Ph.D., Nilanjana Banerjee, Ph.D., Dadong Li, Ph.D., Sameer Malhotra, M.D., Justin Mower, Ph.D., Mudasar Sarwar, Deepika Sharma, Ph.D., Jeffrey C Staples, Ph.D., Jay Sundaram, Sean Yu, Ph.D., Aaron Zhang, Ph.D.

Contribution: Development and validation of clinical phenotypes used to identify study participants and (when applicable) controls.

Genome Informatics & Data Engineering
Jeffrey G Reid, Ph.D., Mona Nafde, M.S., George Mitra, Sujit Gokhale, Andrew Bunyea, Janice Clauer, M.S., MBA, Krishna Pawan Punuru, M.S., Sanjay Sreeram, Gisu Eom, Sujit Gokhale, Benjamin Sultan, M.S., Rouel Lanche, Vrushali Mahajan, Eliot Austin, Koteswararao Makkena, M.S., Sean O'Keeffe, Ph.D., Razvan Panea, Ph.D., Tommy Polanco, Ayesha Rasool, M.S., William Salerno, Ph.D., Xiaodong Bai, Ph.D., Lance Zhang, M.S., Boris Boutkov, Ph.D., Evan Edelstein, Alexander Gorovits, Ph.D., Ju Guan, Ph.D., Lukas Habegger, Ph.D., Alicia Hawes, Olga Krasheninina, M.S., Samantha Zarate, Ph.D., Adam J Mansfield, Evan K Maxwell, Ph.D., Suganthi Balasubramanian, Ph.D., Suying Bao, Ph.D., Kathie Sun, Ph.D., Chuanyi Zhang, Ph.D.

Contribution: Performed and are responsible for analysis needed to produce exome and genotype data, provided compute infrastructure development and operational support, provided variant and gene annotations and their functional interpretation of variants, and conceived and are responsible for creating, developing, and deploying analysis platforms and computational methods for analyzing genomic data.

Analytical Genetics and Data Science
Gonçalo Abecasis, D. Phil., Manuel Allen Revez Ferreira, Ph.D., Joshua Backman, Ph.D., Kathy Burch, Ph.D., Adrian Campos, Ph.D., Lei Chen, Ph.D., Sam Choi, Ph.D., Amy Damask, Ph.D., Liron Ganel, Ph.D., Sheila Gaynor, Ph.D., Benjamin Geraghty, Ph.D., Akropravo Ghosh, M.S., Salvador Romero Martinez, Christopher Gillies, Ph.D., Lauren Gurski, Joseph Herman, D. Phil., Eric Jorgenson, Ph.D., Tyler Joseph, Ph.D., Michael Kessler, Ph.D., Jack Kosmicki, Ph.D., Nan Lin, Ph.D., Adam Locke, Ph.D., Priyanka Nakka, Ph.D., Jonathan Marchini, Ph.D., Karl Landheer, Ph.D., Olivier Delaneau, Ph.D., Maya Ghoussaini, Ph.D., Anthony Marcketta, M.S., Joelle Mbatchou, Ph.D., Arden Moscati,

© 2024 Plym A et al. *JAMA Network Open.*

Ph.D., Aditeya Pandey, Ph.D., Anita Pandit, M.S., Charles Paulding, Ph.D., Jonathan Ross, Carlo Sidore, Ph.D., Eli Stahl, Ph.D., Maria Suciu, Ph.D., Timothy Thornton, Ph.D., Peter VandeHaar, M.S., Sailaja Vedantam, Ph.D., Scott Vrieze, Ph.D., Jingning Zhang, Ph.D., Rujin Wang, Ph.D., Kuan-Han Wu, Ph.D., Bin Ye, Ph.D., Blair Zhang, Ph.D., Andrey Ziyatdinov, Ph.D., Yuxin Zou, Ph.D., Olivier Delaneau, Ph.D., Maya Ghoussaini, Ph.D., Jingning Zhang, Ph.D.

Contribution: Development of statistical analysis plans. QC of genotype and phenotype files and generation of analysis ready datasets. Development of statistical genetics pipelines and tools and use thereof in generation of the association results. QC, review and interpretation of results. Generation and formatting of results for manuscript figures.

Therapeutic Area Genetics
Adolfo Ferrando, M.D., Ph.D., Giovanni Coppola, M.D., Luca A Lotta, M.D., Ph.D., Alan Shuldiner, M.D., Katherine Siminovitch, M.D., Brian Hobbs, M.D., Jon Silver, Ph.D., William Palmer, Ph.D., Rita Guerreiro, Ph.D., Amit Joshi, Ph.D., Antoine Baldassari, Ph.D., Cristen Willer, D. Phil., Sarah Graham, Ph.D., Ernst Mayerhofer, M.D., Jonas Bille Nielsen, Ph.D., Mary Hass, Ph.D., Niek Verwei, Ph.D.1j, George Hindy, Ph.D., Jonas Bovijn, M.D., Tanima De, Ph.D., Parsa Akbari, Ph.D., Luanluan Sun, Ph.D., Olukayode Sosina, Ph.D., Arthur Gilly, Ph.D., Peter Dombos, Ph.D., Juan Rodriguez-Flores, Ph.D., Moeen Riaz, Ph.D., Manav Kapoor, Ph.D., Gannie Tzoneva, Ph.D., Momodou W Jallow, Ph.D., Anna Alkelai, Ph.D., Giovanni Coppola, M.D., Ariane Ayer, Veera Rajagopal, M.D., Sahar Gelfman, Ph.D., Vijay Kumar, Ph.D., Jacqueline Otto, Ph.D., Neel Parikshak, M.D., Aysegul Guvenek, Ph.D., Jose Bras, Ph.D., Silvia Alvarez, Ph.D., Jessie Brown, Ph.D., Jing He, Ph.D., Hossein Khiabanian, Ph.D.

Contribution: Development of study design and analysis plans. Development and QC of phenotype definitions. QC, review, and interpretation of association results.

Research Program Management & Strategic Initiatives
Lyndon J Mitnaul, Ph.D., Marcus B Jones, Ph.D., Esteban Chen, M.S., Michelle G LeBlanc, Ph.D., Jason Mighty, Ph.D., Nirupama Nishtala, Ph.D., Nadia Rana, Ph.D., Jennifer Rico-Varela, Ph.D., Jaimee Hernandez.

Contribution: Contributed to the management and coordination of all research activities, planning and execution, managed the review of the project.

Senior Partnerships & Business Operations
Alison Fenney, Ph.D., MBA, Jody Hankins, Ph.D., MBA, Samuel Hart, J.D.

Contribution: Contributed to the management, planning, execution, and negotiation of new and existing agreements.

Business Operations & Administrative Coordinators
Ann Perez-Beals, Gina Solari, Jaimee Hernandez, Johannie Rivera-Picart, Michelle Pagan, Sunilbe Siceron.

Contribution: coordinate all administrative activities with internal stakeholders and external collaborators.

© 2024 Plym A et al. *JAMA Network Open.*

eAppendix 2. Supplementary Acknowledgements

CRUK and PRACTICAL consortium

This work was supported by the Canadian Institutes of Health Research, European Commission's Seventh Framework Programme grant agreement n° 223175 (HEALTH-F2-2009-223175), Cancer Research UK Grants C5047/A7357, C1287/A10118, C1287/A16563, C5047/A3354, C5047/A10692, C16913/A6135, and The National Institute of Health (NIH) Cancer Post-Cancer GWAS initiative grant: No. 1 U19 CA 148537-01 (the GAME-ON initiative).

We would also like to thank the following for funding support: The Institute of Cancer Research and The Everyman Campaign, The Prostate Cancer Research Foundation, Prostate Research Campaign UK (now PCUK), The Orchid Cancer Appeal, Rosetrees Trust, The National Cancer Research Network UK, The National Cancer Research Institute (NCRI) UK. We are grateful for support of NIHR funding to the NIHR Biomedical Research Centre at The Institute of Cancer Research, The Royal Marsden NHS Foundation Trust, and Manchester NIHR Biomedical Research Centre. The Prostate Cancer Program of Cancer Council Victoria also acknowledge grant support from The National Health and Medical Research Council, Australia (126402, 209057, 251533, , 396414, 450104, 504700, 504702, 504715, 623204, 940394, 614296,), VicHealth, Cancer Council Victoria, The Prostate Cancer Foundation of Australia, The Whitten Foundation, PricewaterhouseCoopers, and Tattersall's. EAO, DMK, and EMK acknowledge the Intramural Program of the National Human Genome Research Institute for their support.

Genotyping of the OncoArray was funded by the US National Institutes of Health (NIH) [U19 CA 148537 for ELucidating Loci Involved in Prostate cancer SuscEptibility (ELLIPSE) project and X01HG007492 to the Center for Inherited Disease Research (CIDR) under contract number HHSN268201200008I]. Additional analytic support was provided by NIH NCI U01 CA188392 (PI: Schumacher).

Research reported in this publication also received support from the National Cancer Institute of the National Institutes of Health under Award Numbers U10 CA37429 (CD Blanke), and UM1 CA182883 (CM Tangen/IM Thompson). The content is solely the responsibility of the authors and does not necessarily represent the official views of the National Institutes of Health.

Funding for the iCOGS infrastructure came from: the European Community's Seventh Framework Programme under grant agreement n° 223175 (HEALTH-F2-2009-223175) (COGS), Cancer Research UK (C1287/A10118, C1287/A 10710, C12292/A11174, C1281/A12014, C5047/A8384, C5047/A15007, C5047/A10692, C8197/A16565), the National Institutes of Health (CA128978) and Post-Cancer GWAS initiative (1U19 CA148537, 1U19 CA148065 and 1U19 CA148112 - the GAME-ON initiative), the Department of Defence (W81XWH-10-1-0341), the Canadian Institutes of Health Research (CIHR) for the CIHR Team in Familial Risks of Breast Cancer, Komen Foundation for the Cure, the Breast Cancer Research Foundation, and the Ovarian Cancer Research Fund.

BPC3

The BPC3 was supported by the U.S. National Institutes of Health, National Cancer Institute (cooperative agreements U01-CA98233 to D.J.H., U01-CA98710 to S.M.G., U01-CA98216 to E.R., and U01-CA98758 to B.E.H., and Intramural Research Program of NIH/National Cancer Institute, Division of Cancer Epidemiology and Genetics).

CAPS

CAPS GWAS study was supported by the Cancer Risk Prediction Center (CRisP; www.crispcenter.org), a Linneus Centre (Contract ID 70867902) financed by the Swedish Research Council, (grant no K2010-70X-20430-04-3), the Swedish Cancer Foundation (grant no 09-0677), the Hedlund Foundation, the Soederberg Foundation, the Enqvist Foundation, ALF funds from the Stockholm County Council. Stiftelsen Johanna Hagstrand och Sigfrid Linner's Minne, Karlsson's Fund for urological and surgical research.

PEGASUS

PEGASUS was supported by the Intramural Research Program, Division of Cancer Epidemiology and Genetics, National Cancer Institute, National Institutes of Health.

© 2024 Plym A et al. *JAMA Network Open.*
