# Supplementary material for: Early Prostate Cancer Deaths Among Men With Higher vs Lower Genetic Risk
Source: JAMA Netw Open. 2024 Jul 3;7(7):e2420034. doi: 10.1001/jamanetworkopen.2024.20034 (PMC11222990; doi:10.1001/jamanetworkopen.2024.20034)
Supplement: Supplement 2. — Data Sharing Statement [file jamanetwopen-e2420034-s002.pdf]

Data Sharing Statement

Plym. Early Prostate Cancer Deaths Among Men With Higher vs Lower Genetic Risk. *JAMA Netw Open*. Published July 03, 2024. doi:10.1001/jamanetworkopen.2024.20034

Data
Data available: Yes
Data types: Deidentified participant data
How to access data: Data are available for researchers through a project proposal for the Health Professionals Follow-up Study (https://sites.sph.harvard.edu/hpfs/for-collaborators) and the Malmö Diet and Cancer Study (https://www.malmo-kohorter.lu.se/uttag/uttagsansokningar-mkcmfm).
When available: With publication

Supporting Documents
Document types: None

Additional Information
Who can access the data: Researchers whose proposed use of the data has been approved.
Types of analyses: For approved research purposes.
Mechanisms of data availability: After project approval.
